# Health Care Professionals’ Experiences With Using Information and Communication Technologies in Patient Care During the COVID-19 Pandemic: Qualitative Study

**DOI:** 10.2196/53056

**Published:** 2024-05-28

**Authors:** Carly A Cermak, Heather Read, Lianne Jeffs

**Affiliations:** 1 Science of Care Institute Sinai Health Toronto, ON Canada; 2 Lunenfeld-Tanenbaum Research Institute Sinai Health Toronto, ON Canada; 3 Institute of Health Policy, Management and Evaluation University of Toronto Toronto, ON Canada

**Keywords:** COVID-19, information and communication technology, health care provider experiences, web-based care, interview

## Abstract

**Background:**

The COVID-19 pandemic acted as a catalyst for the use of information and communication technology (ICT) in inpatient and outpatient health care settings. Digital tools were used to connect patients, families, and providers amid visitor restrictions, while web-based platforms were used to continue care amid COVID-19 lockdowns. What we have yet to learn is the experiences of health care providers (HCPs) regarding the use of ICT that supported changes to clinical care during the COVID-19 pandemic.

**Objective:**

The aim of this paper was to describe the experiences of HCPs in using ICT to support clinical care changes during the COVID-19 pandemic. This paper is reporting on a subset of a larger body of data that examined changes to models of care during the pandemic.

**Methods:**

This study used a qualitative, descriptive study design. In total, 30 HCPs were recruited from 3 hospitals in Canada. One-on-one semistructured interviews were conducted between December 2022 and June 2023. Qualitative data were analyzed using an inductive thematic approach to identify themes across participants.

**Results:**

A total of 30 interviews with HCPs revealed 3 themes related to their experiences using ICT to support changes to clinical care during the COVID-19 pandemic. These included the use of ICT (1) to support in-person communication with patients, (2) to facilitate connection between provider to patient and patient to family, and (3) to provide continuity of care.

**Conclusions:**

HCP narratives revealed the benefits of digital tools to support in-person communication between patient and provider, the need for thoughtful consideration for the use of ICT at end-of-life care, and the decision-making that is needed when choosing service delivery modality (eg, web based or in person). Moving forward, organizations are encouraged to provide education and training on how to support patient-provider communication, find ways to meet patient and family wishes at end-of-life care, and continue to give autonomy to HCPs in their clinical decision-making regarding service delivery modality.

## Introduction

The health care workforce had to quickly adapt to the COVID-19 pandemic, with health systems grappling with the provision of COVID-19 care at the same time as non-COVID-19 care. Restrictions to reduce the spread of COVID-19 put an additional strain on the health care system. Health care providers (HCPs) were left to problem-solve how to continue providing compassionate, connected care among layers of personal protective equipment and visitor restrictions. Fortunately, the COVID-19 pandemic was a catalyst for digital health to support the ongoing response to the COVID-19 pandemic, with web-based care emerging as the primary innovation of information and communication technology (ICT) used in medical care [1,2]. Uses of ICT in medical care include remote consultations, digital noninvasive care, and digital platforms for data sharing [3].

ICT played an important role in supporting changes to clinical care within inpatient and outpatient health care settings. Within inpatient settings, ICT was integral in maintaining connectivity between patients, families, and providers when changes to visitor policies were implemented [4]. For example, the use of mobile devices and tablets allowed for connection between patient and family and supported knowledge transfer between provider and family [5]. Within outpatient settings, ICT was integral in continuing care when COVID-19 lockdown restrictions limited in-person visits [1]. For example, videoconference and telemedicine services (ie, web-based care) emerged as a platform for providers to use to allow for remote care [1]. In both facets, ICT facilitated connection, acting as an essential link between patients, families, and providers. However, we have yet to learn of HCPs’ experiences in using ICT to support clinical care.

Learning from the experiences of HCPs’ use of ICT will offer valuable insights into how innovative uses of ICT might continue to be used in inpatient and outpatient health care settings moving forward. From here, uses of ICT can inform organizational leadership of the systems or processes that may require further investigation to support ICT use in clinical care in a postpandemic world. The main objective of the study was to examine changes to models of care during the pandemic from the perspectives of HCPs, implementation team members, and leaders across 3 Canadian hospitals. For this paper, we report on a storyline that emerged from this work to describe the experiences of HCPs’ use of ICT that supported changes to clinical care during the COVID-19 pandemic.

## Methods

### Study Design

This qualitative descriptive study was undertaken from March 2022 to June 2023 to understand changes to models of care during the COVID-19 pandemic through the experiences of HCPs, implementation team members, and leaders across 3 hospitals in Canada. This paper is reporting on a subset of data related to HCPs’ experiences of using ICT in supporting changes to clinical care, drawn from the larger study that explored changes to models of care that took place during the COVID-19 pandemic. The reporting of this study was guided by the Standards for Reporting Qualitative Research [6].

### Sampling and Participant Recruitment

In total, 30 HCPs were recruited from critical care, inpatient, and ambulatory services across 3 hospitals in Canada. A purposeful sampling strategy was used where recruiting took place in organizations that were known to have been affected by COVID-19 restrictions and policies. Site leads at participating institutions disseminated study information to HCPs (eg, nurses, physicians, and allied health disciplines) working within their respective health care organizations. From here, interview participants self-referred to this study. Inclusion criteria included current employment as an HCP working at the health care organization over the course of the pandemic and postpandemic recovery.

### Data Collection

One-on-one, semistructured interviews were conducted by members of the research team (Kang Kang Margolese, Marina Morris, Lily Zeng, Marie Oliveira, Adebisi Akande, HR, Frances Bruno, or CAC) between December 2022 and June 2023. Demographic information, including age, gender, ethnicity, health discipline, time in profession, time in organization, and time in current role, was collected from all participants before the interview to ensure diversity within the sample. An interview guide was developed by the research team that explored the following five areas: (1) changes to care (eg, “What was your role like before the pandemic? How did care change over the course of the last 3 years?”), (2) provisions of care (eg, “What did you/your team start/stop doing? How did you prioritize care?”), (3) emotions (eg, “How did care change feel for you/your team? What supports were available to you?”), (4) implementation and evaluation (eg, “How were changes implemented and evaluated?”), and (5) lessons that were learned or future recommendations.

Data collection was completed by nonclinical research staff (Kang Kang Margolese, Marina Morris, Lily Zeng, Adebisi Akande, and HR) and clinical research staff (Marie Oliveira, Frances Bruno, and CAC). Data collection was concluded when saturation of themes was reached, meaning that limited new insights emerged from existing themes with the collected data sample [7]. The interviews were conducted via either a videoconferencing platform or in person and were approximately 45 to 60 minutes in length.

### Ethical Considerations

Ethics was formally reviewed and approved by Sinai Health’s Research Ethics Board (REB# 22-0153-E), as well as at each participating site: Sunnybrook Health Sciences Centre (REB# 5571) and Providence Health Care (REB# H22-02792). Participants were informed that participation in this study was completely voluntary and that they could withdraw from the study at any time without penalty. Verbal informed consent was obtained before the start of the interviews, and participants were given an electronic gift card in recognition of their time. The honorarium for participants was CAD $20 (US $26.4). Demographic information was collected from all participants before the interview. These data were anonymized and stored separately from the transcripts, which were deidentified and stored on a secure server.

### Data Analysis

The research design was conceived within an interpretivist paradigm, where the researchers’ purpose was to gather insight into how clinical care changed during the COVID-19 pandemic through the learning of the experiences of participants [8]. Interviews were analyzed using an inductive thematic analysis approach, which included openly coding line by line to organize data in a meaningful, systematic way; examining the codes to identify themes; and reviewing the themes [9]. Specifically, the entire research team openly coded a small group of interviews (n=3) independently, line by line, and then met as a group to review codes, discuss themes, and develop an initial codebook through consensus. From here, the research team coded the bulk of the interviews in pairs, meeting as needed to ensure the reliability of coding, using the primary investigator (LJ) to triangulate and resolve any discrepancies as needed.

Reflexivity was demonstrated through regular debriefs of interviews and a review of the codebook at 1- to 2-week intervals during the coding process. Primary adjustments were additions of new codes as interviews were collected from new participant subgroups. For example, the initial codebook was derived from nurse interviews, and new codes were required as the project expanded into allied health disciplines. Codes that related to HCPs’ experiences of ICT included disciplinary changes, technical changes and innovations, improvisation, problem-solving, tools, and technology recommendations. NVivo software (QSR International) was used to facilitate the cross-synthesis analysis. As a final step of analysis to ensure saturation and methodological rigor, the primary investigator for the study (LJ) reviewed the emergent coding schema with the original transcripts.

## Results

### Participant Characteristics

A total of 30 participants (site A: n=4, site B: n=14, and site C: n=12) described their experiences of how ICT supported changes to clinical care. Table 1 presents the demographic characteristics. Themes generated from participants included the use of ICT (1) for supporting in-person communication with patients; (2) for enabling connection between patients, providers, and families; and (3) for providing continuity of care amid COVID-19 restrictions.

**Table 1:** Demographic characteristics, HCP^a^ roles, and time in the profession of study participants (N=30).

Characteristic		Values
**Age range (years), n (%)**		
	26-30	3 (10)
	31-35	3 (10)
	36-40	3 (10)
	41-45	9 (30)
	46-50	3 (10)
	51-55	6 (20)
	56-60	1 (3)
	61+	2 (7)
**Sex, n (%)**		
	Female	30 (100)
	Male	0 (0)
**Ethnicity^b^, n (%)**		
	Asian	4 (13)
	Canadian	6 (20)
	European	2 (7)
	Hispanic	1 (3)
	White	15 (50)
	Mixed	2 (7)
**HCP disciplines, n (%)**		
	Nursing	6 (20)
	Social work	8 (27)
	Music therapy	2 (7)
	Physiotherapy	3 (10)
	Recreation therapy	2 (7)
	Occupational therapy	2 (7)
	Spiritual care	3 (10)
	Dietetics	3 (10)
	Psychology	1 (3)
**Time (years), mean (SD)**		
	In profession	16.84 (8.59)
	At organization	14.23 (8.82)
	In current role	10.95 (8.20)

^a^HCP: health care provider.

^b^Participant self-identified; categories were not provided.

### Supporting In-Person Communication With Patients

Participants described how tablets supported in-person communication to mitigate the impact that personal protective equipment (PPE) had on verbal interactions with patients. PPE such as masks, Plexiglas, and visors posed challenges in communicating effectively, particularly for patients who were hard of hearing or who had difficulties with comprehension. Efforts to support communication were essential as communication breakdowns created confusion for the patients with detrimental consequences:

And so when talking to elderly people, when they can’t read your lips or when they can’t really hear you through three layers of protective equipment, they get very confused and multiple confusing events leads to possible more agitation and agitation leads to an automatic write-off from a lot of health care providers as to a reason why not to provide a certain person with care.

Site B, 01, physiotherapist

Participants described coming up with innovative ways to facilitate communication amid the layers of PPE, with tablets and phones used to break down communication barriers. Applications such as speech to text allowed live transcription of providers’ speech, which can be used as a tool to support comprehension for patients who were hearing impaired. Further, speech-to-text applications provided patients and families a model of how this tool can be used to support communication outside of the hospital setting:

And so, this [iPads] has been a huge help...it helps people, patients who haven’t heard of this...they go home with a brand-new strategy that makes their daily life so much easier.

Site C, 08, social worker

In addition to using tablets to support communication with patients who were hard of hearing, participants also expressed the value of using tablets for translation services for patients who did not speak English. Benefits included the convenience of dialing translation services from an iPad:

We have translation services on them [iPads]...which has been so, so wonderful to have to just go into someone’s room who doesn’t speak English...And just call up this interpretation service, have a human being there and that was really a key.

Site C, 29, spiritual health practitioner

Challenges surfaced when both a videoconferencing platform and translation services were required—specifically, the difficulties in handling 2 ICT tools simultaneously and the need to prioritize videoconferencing all the while hoping that family members were relaying information correctly:

...you can’t hold a Zoom, you know, iPad and then hold a translator phone to it, you know what I mean? So then it became family trying to find someone at their end who could relay information.

Site B, 13, occupational therapist

### Enabling Connection Between Patients, Providers, and Families

Participants described how digital devices facilitated the connection between provider to family and provider to patient during visitor restrictions. This included using phones and iPads to connect families to their loved ones in hospitals, especially at end-of-life care. Participants also described that providing a digital connection to families at end-of-life care was a service that could help families move through the grief process.

...we facilitated a FaceTime and all kinds of video calls for people to be able to talk to their loved ones. And even to their religious leaders in certain cases...Families were not able to be with a loved one when they were dying…we were a bridge between them.

Site B, 07, spiritual care

...we recorded a memorial service that was generic and was put up on YouTube and we could send the link...And so many people just didn’t have the needed ritual to move through grief. And that was something that we could give them and that was—we received so much good feedback and gratitude for that.

Site C, 29, spiritual health practitioner

While there were benefits of tablet use to connect families to patients at end-of-life care, a digital connection created an internal struggle for HCPs as they witnessed the lack of physical touch and difficulties in accommodating end-of-life rituals:

I feel like I struggled when I had to use an iPad to connect patients to family members and it could be in a very vulnerable situation, like a patient was dying, he doesn’t speak English, the daughter’s on the iPad, she’s crying, she can’t hold her dad, can’t hold his hand...I think we have to recognize that...there is a rite of passage before somebody dies. There are certain steps for religious people and families that need to happen to honour a dying body for them to move on to wherever that place is...So anointing, communion, confession. Those are not things that are amenable to a Zoom method.

Site B, 12, nursing

Further, participants expressed the challenges with navigating the frequency of communication between patient and family, such as balancing family requests with staffing resources within the hospital:

...when you had multiple family members who each wanted their turn to visit once a week. Well, you know, you don’t have staff to be able to support five Facetimes per resident. So, we started to have to limit it and say...like two Facetimes a week for a family, or for a resident...So, that was a challenge.

Site B, 05, social worker

### Providing Continuity of Care

Participants described how the use of videoconferencing platforms such as Zoom (Zoom Video Communications) enhanced communication between providers and families, such as when needing to provide medical updates or discharge recommendations. Zoom provided accessible options for patients with hearing or comprehension challenges using closed captioning. Furthermore, Zoom enabled more efficient and faster communication between the care team and family, rather than being faced with the complexities of coordinating schedules of team members and families who may be coming in from out of town:

It [Zoom] optimized our efficiency for delivering family meetings...the specialist physicians were able to attend more of these family meetings than in the past, because of the ability to attend virtually. And then, more family were able to attend than...in the past. And it was able to happen faster because we could do it virtually versus waiting several days for a family member to arrive from another city.

Site B, 13, occupational therapist

Participants also expressed the benefits of web-based care for patient access, particularly for patients with mobility challenges or lack of transportation:

I can actually say that shift [to virtual] was very positive because...it actually eliminated some of the concerns my patients have about transportation, or ways that they’re able to get out there, be it because of their physical impairment post-operation. Or simply just because they don’t have the resources to get transit for whatever reason.

Site A, 23, social worker

Further, some participants expressed how web-based care positively changed clinical practice for counseling services:

And from all the patients I’ve intervened with...I’d say .01% want to come in person...I find that on Zoom you can sort of see the environment they’re in...I think that COVID has revolutionized social work intervention...I only have good things to say about it. COVID has opened up a whole new world for counseling.

Site B, 15, social worker

Web-based care was not without its challenges. Clinicians described that greater access to care increased referrals from patients who would historically not come for in-person treatment, particularly for mental health services:

...we found that we were getting more referrals from ... all these different patients who would have not been able to come to hospital to do in-person groups...people with anxiety disorders, like agoraphobia. People who had not seen—have difficulty going outside the house.

Site A, 16, nursing

...the workload increased enormously, and was impossible to keep up with because before people had to come in to [the hospital] to see me so that actually restricted the number of people that I could see to people who lived in [the city], or in some neighbouring communities. At times, people would come in and come drive like 90 to 120 minutes to come and see me but due to Covid, when we shifted to online therapy...now, everybody in [the province] had access to me who were part of these programs...many people wanted to see the psychologist because they wouldn’t have to drive in.

Site C, 16, psychologist

Consequently, participants described that more visits over Zoom led to greater fatigue as a result of having to simultaneously navigate Zoom and in-person teaching, resulting in a reduction in group therapy frequency:

We noticed for us clinicians we were just getting so fatigued that it was just too much. Because running a group in-person, and running it over Zoom is very, very different. You’re staring at a screen, you’re looking at all the faces in the room. You’re trying to navigate the PowerPoint, there’s a lot of things happening simultaneously, that when we were doing four groups a week we just noticed this is not sustainable for us. So we had to shift it to three groups. So one less group a week. So I think that’s a huge change in terms of provision of care.

Site A, 16, nursing

In terms of providing clinical care, clinicians described the challenges of conducting a physical assessment or providing counseling treatment via Zoom or by phone:

We do some physical examination. So it’s hard just to understand the status just by phone, even if you ask them “Any swelling?” Then they say no but actually they have, so the knowledge may not be there.

Site C, 10, registered dietician

...in Zoom it’s very limited and you mostly see the face. Right? You don’t see what the person is doing with their hands, arms, with their legs, with their feet.

Site B, 07, spiritual care practitioner

It’s just something about being in the same room with someone when their emotions are high that you don’t actually have to do anything in particular, but just the calming presence makes a difference. I think that people get some of that on Zoom...I don’t know how similar or different, but I’m just assuming that it’s probably a bit watered down...Whereas if I was just in the room, I think just being quiet with the person would be enough and might be even better at times.

Site C, 16, psychologist

Finally, clinicians described the challenges of using web-based care when working with older patients due to limited experiences with technology or cognitive impairments. Interestingly, some participants felt that the reliance on web-based care reduced the attendance of older populations who were not familiar with the technology.

...our average age is 97, they’re not tech savvy, they’re not necessarily understanding, comprehending, you know, that, you know, as we would understand that you can actually talk to someone who’s not present here, but it’s in the same time...So, I would call it, you’re having a video call. I try and explain it’s that, you’re having a video telephone call. And then, they just think they’re looking at a television, you know, and they’re just watching kind of a show and stuff.

Site B, 10, recreation therapist

Some of our clients—some people with dementia don’t understand...either they don’t recognize themselves, or they get agitated by the sight of themselves—so having the person facilitating the Zoom understand how to turn off the view that you can see yourself, was important...I think I lost a number of older spouses that used to come to the group, because they...had difficulty understanding the technology, or just their digital literacy, or access to technology wasn’t that great. So currently...and interestingly, that has changed the demographic of people who are coming in my Caregiver Group.

Site B, 08, social worker

## Discussion

### Principal Findings

The aim of this qualitative descriptive study was to describe the experiences of HCPs in how ICT supported changes to clinical care during the COVID-19 pandemic. Participant narratives revealed 3 key findings: the benefits of digital tools to support in-person communication between patient and provider, the need for thoughtful consideration for the use of ICT at end-of-life care, and the support for the continued use of web-based care, when appropriate. We discuss HCPs’ experiences as they relate to the literature and provide recommendations for health care organizations that can make use of ICT in a more collaborative way while reflecting on patient and family values.

### Supporting In-Person Communication With Patients

Communication between patients and providers is essential for quality care and for reducing preventable adverse medical events [10]. Patients who have been appropriately supported in their communication have reported to be more satisfied in their hospital stay [11]. Devices to assist with communication, more commonly referred to as alternative augmentative communication (AAC), have existed in health care for decades. AAC is an intervention approach for individuals who require added support (augmentative) or a replacement (alternative) for their communication [12]. AAC can be low technology such as communication boards or pictures or high technology such as communication systems on iPads and speech-generation devices and can be used for a short or long period of time depending on the individual’s communication needs [12].

The COVID-19 pandemic spawned a rapid adoption of digital tools such as tablets, which became an available tool to reduce communication barriers experienced with mask-wearing when speaking to patients and families and allow for participation in conversation. Additionally, tablets enabled access to video language interpretation for patients who were mechanically ventilated and awake [13], a unique example of reducing language barriers when families were not able to be present for interpretation. However, participant narratives using digital tools within acute care and rehabilitation contrast the literature describing the experiences of patients and families in the intensive care unit. In the intensive care unit, HCPs and families reported barriers to the implementation of communication supports, particularly for patients who were mechanically ventilated and awake [14]. Nurses reported feeling inadequate and frustrated in trying to support patients [14], whereas families reported frustration with communication breakdowns, inconsistent availability of tools, and insufficient training by the HCP [15]. Patients described being mechanically ventilated as a vulnerable, lonely, and fearful experience [15], particularly as verbal communication was not an option.

The collective experiences of nurses, families, and patients emphasize the impact that a lack of communication supports can have at the bedside. Further, the experiences of nurses, families, and patients shed light on the education and training that is needed for successful patient-provider communication to support participation in conversation, particularly for patients on mechanical ventilation. Reports from speech-language pathologists working with patients who are critically ill revealed positive patient-provider communication outcomes when there was nurse collaboration and readily available communication supports at the bedside [13]. Thus, the experiences of patients, families, and HCPs highlight the integral role that leadership and hospital policies play in prioritizing communication access, tool availability, and organizational-wide training [13,16]. For system-level change, it is recommended that hospital leaders develop regular staff training on communication supports led by professionals with expertise in this area such as speech-language pathologists [14]. For increased awareness on the importance of communication supports in health care, it is recommended that education on patient-provider communication starts as early as the undergraduate and postgraduate level for health discipline (ie, clinical) programs [14].

### Enabling Connection Between Patients, Providers, and Families

Videoconferencing tools have been used to connect loved ones for over a decade and have been shown to have positive psychosocial outcomes for nursing home residents when used as an addition to in-person family visits [17]. Specifically, older residents in nursing homes who received videoconferencing visits with family in addition to in-person family visits had a greater mean change in baseline depressive symptoms and feelings of loneliness when compared to older residents who had in-person visits only [17]. During the pandemic, however, videoconferencing tools and digital devices were used as a substitute for in-person visits due to visitor restrictions imposed by the COVID-19 pandemic. Although this enabled a connection between patient and family, the reduced frequency of family connections created tensions between both HCPs and family members.

Similar tensions were described by HCPs in the United Kingdom including communicating devastating news to relatives without having ever met them in person and the moral dilemma of what is “best” end-of-life care versus what could be offered given the COVID-19 restrictions [18]. Further, clinicians in Canada reported that web-based visits at end-of-life care prevented meaningful conversations typically had between family members at the bedside [19]. One physician described the importance of family connection in end-of-life care: “I’m now convinced that family members at the bedside improves patients’ ability to get better” [19]. The experiences of bereaved relatives aligned with the internal conflicts of HCPs in the United Kingdom: families wanted frequent communication that was easy to understand, one last chance to say goodbye through physical touch, and speaking to their loved one at bedside [20]. Similarly in Canada, HCPs, patients, and families all felt that restrictive acute care visitor policies impacted the safety and quality of care, mental health of everyone involved, families as partners in care, and communication and advocacy [4].

Although COVID-19 visitor restrictions have lifted, the experiences described by clinicians and families highlight the considerations needed for a positive, meaningful, end-of-life experience. One example of an organizational-wide intervention for end-of-life care includes the 3 Wishes Project (3WP), an intervention that gathers 3 wishes from the patient and family to help personalize and humanize end-of-life care [21]. The 3WP has demonstrated a positive impact on families and clinicians; families had a significantly higher rating of emotional and spiritual support than families who did not receive the 3WP [22], while clinicians reported greater morale and collaboration in helping families move toward acceptance [23]. Further, the 3WP has shown to build capacity for compassion at the organization level by facilitating collective noticing, feeling, and responding [24]. In other words, the implementation of 3WP creates system-level processes and structures to facilitate compassionate care while promoting the connection between patients, families, and HCPs [24]. Thus, while the use of digital devices will likely continue to be a complement to care [25], it is important that organizations encourage collective, compassionate care to meet the wishes of patients and families.

### Providing Continuity of Care

Literature describing the benefits and challenges of web-based care aligned with participant narratives. Benefits included faster access to care, greater efficiency, and improved convenience for patients [26]; challenges included conducting assessments without the ability to complete in-person physical examinations [26] and offering web-based care to patients with poor digital literacy [27-29]. What was unique to this study’s findings was the increase in referral rates with the implementation of web-based care. Two reasons for an increase in referrals as described by participants included greater access for patients with significant mental health needs who otherwise would not come in for services and greater access for patients living far away from the hospital. Consequently, more referrals increased the workload of HCPs, demonstrating the dichotomy between patient access to care and provider workload. This emphasizes the considerations needed to balance clinician workload with patient preference of service modality as organizations move toward hybrid models of care [25].

A recent US study examined patient preference for service modality for nonurgent care and found that when out-of-pocket costs were not a factor, slightly more than half of the sample (53%) preferred in-person visits to web-based care, while one-fifth (21%) preferred web-based and one-quarter (26%) had no preference or did not know what they preferred [30]. For individuals who had video visit experience, this was associated with their preference for video visits [30]. A closer look at demographic factors revealed that those who did not feel that video calls had a role in their medical care were generally older people, who lived rurally, and who had a lower income and educational level [30]. Conversely, patients who were younger and had a higher income and education were more likely to choose a video visit over in-person care [30]. While choice of service modality may be an option for nonurgent care moving forward, some populations may not have the same ability to choose. Rather, it is up to the HCP to decide whether web-based care is appropriate.

HCPs, such as psychiatrists, who work with patients with significant mental health disorders have described the role that contextual factors contribute to decision-making of service modality [31]. Contextual factors in decision-making included if an in-person visit provided greater therapeutic benefit than a web-based visit, if a general examination was needed, if there were caregivers nearby who could provide information, if insight into the living environment was necessary, and if safety resources were required for in-person visits [31]. There was no consensus among psychiatrists on the mental health conditions that would best be served, as some respondents felt web-based care offered unique benefits such as improved patient safety and reduced likelihood of escalation [31]. Taken together, a combination of factors will need to continue to be considered for service delivery modality moving forward, such as patient preference, nature of service provided, and technology literacy. Furthermore, thoughtful planning for the accessibility of technology use for underserved populations will likely be an element of consideration for the field of health care [32].

### Limitations

First, this study is limited to the experiences of the HCP from urban hospitals in Ontario and British Columbia and may not be transferable to the full scope of pandemic hospital worker experiences across the globe. Consequently, there may have been uses of ICT that happened during the pandemic that were particularly novel or interesting but may not have been captured due to the nature of this qualitative study. Second, participants were given an electronic gift card after the interview in recognition of their time, which may have impacted self-referral into the study. Third, there were several research team members involved in interviews, which may have impacted the depth of information provided by the participants across interviews.

### Conclusions

Experiences from HCP highlight the uses of ICT to support changes to clinical care during the pandemic. The use of digital tools supported patient-provider communication, enabled a connection between patients and families at end-of-life care, and provided continuity of care amid COVID-19 lockdowns. Moving forward, organizations are encouraged to provide education and training on how to support patient-provider communication in clinical care; find ways to implement collaborative, compassionate, end-of-life care; and continue to give autonomy to HCPs in their clinical decision-making regarding service delivery modality.

## Abbreviations

3WP: 3 Wishes Project
AAC: alternative augmentative communication
HCP: health care provider
ICT: information and communication technology
PPE: personal protective equipment

## Data Availability

An aggregate summary of data generated and analyzed during this study are included in this published manuscript. Individual data transcripts cannot be publicly shared because of confidentiality.
